# Ferric Carboxymaltose Therapy Improves the Index of Cardiac Electrophysiological Balance in Iron-Deficient Heart Failure with Reduced Ejection Fraction

**DOI:** 10.3390/medicina62091700

**Published:** 2026-09-04

**Authors:** Çağatay Tunca, Veysel Ozan Tanık, Etkin Elifoğlu, Betül Akıncıoğlu, Furkan Enes Solak, Alperen Taş, Hacı Ali Kürklü, Vedat Hekimsoy, Mehmet Taha Özkan, Meltem Altınsoy, Özge Kurmuş Ferik, Bülent Özlek

**Affiliations:** 1 Department of Cardiology, Ankara Etlik City Hospital, 06170 Ankara, Türkiye; md.tunca@gmail.com (Ç.T.); drozantanik@gmail.com (V.O.T.); etkinelifoglu@gmail.com (E.E.); betulakincioglu@hotmail.com (B.A.); fes.solak@gmail.com (F.E.S.); hacialikurklu@hotmail.com (H.A.K.); vhekimsoy@yahoo.com (V.H.); mehmettahaozkan@gmail.com (M.T.Ö.); mdmeltemaltinsoy@gmail.com (M.A.); ozge_kurmus@yahoo.com (Ö.K.F.); 2 Department of Cardiology, Kırşehir Training and Research Hospital, 40100 Kırşehir, Türkiye; alperentas555@hotmail.com; 3 Department of Cardiology, School of Medicine, Muğla Sıtkı Koçman University, 48000 Muğla, Türkiye

**Keywords:** ferric carboxymaltose, heart failure with reduced ejection fraction, index of cardiac electrophysiological balance, iron deficiency, ventricular repolarization

## Abstract

*Background and Objectives:* Iron deficiency (ID) is common in heart failure with reduced ejection fraction (HFrEF) and is associated with adverse clinical outcomes. Although intravenous ferric carboxymaltose (FCM) improves functional status, its effects on ventricular electrophysiological indices remain incompletely characterized. This study evaluated short-term changes in the corrected index of cardiac electrophysiological balance (iCEBc) and conventional electrocardiographic repolarization parameters after FCM administration. *Materials and Methods:* This retrospective single-center pre–post study included 92 clinically stable patients with HFrEF and ID who received intravenous FCM. Standard 12-lead electrocardiograms and laboratory parameters were evaluated at baseline and 30 ± 5 days after treatment. The primary outcome was the within-patient change in iCEBc, while secondary outcomes included conventional ventricular repolarization indices. *Results:* Following FCM treatment, ferritin, transferrin saturation, and hemoglobin increased significantly (all *p* < 0.001). iCEBc decreased significantly (4.33 ± 1.04 vs. 4.04 ± 0.82, *p* = 0.016). Significant reductions were also observed in QTc, JTc, JT, Tp-e, Tp-e/QT, Tp-e/QTc, frontal QRS–T angle, and QT, whereas QRS duration remained unchanged. Changes in iron indices were not associated with changes in iCEBc. *Conclusions:* In this uncontrolled pre–post cohort, intravenous FCM was associated with favorable short-term changes in several electrocardiographic repolarization indices. However, because anemia correction occurred concurrently and no control group or clinical arrhythmic outcomes were available, these findings should be considered exploratory and hypothesis-generating rather than evidence of an antiarrhythmic effect.

## 1. Introduction

Heart failure with reduced ejection fraction (HFrEF) remains a major contributor to cardiovascular morbidity and mortality worldwide despite substantial advances in guideline-directed medical therapy [1]. In addition to progressive pump failure, ventricular arrhythmias and sudden cardiac death remain important contributors to adverse outcomes in this population [2]. Therefore, identifying potentially modifiable factors associated with changes in myocardial electrical properties remains clinically relevant.

Iron deficiency (ID) is one of the most prevalent comorbidities in patients with HFrEF, affecting approximately half of patients irrespective of anemia status [3]. ID is associated with impaired exercise capacity, reduced quality of life, increased heart failure hospitalizations, and higher mortality [4]. Randomized controlled trials have demonstrated that intravenous iron therapy improves symptoms, functional capacity, and quality of life in patients with HFrEF and ID [5,6], supporting its inclusion in contemporary heart failure management guidelines [7]. However, the association between ferric carboxymaltose (FCM) administration and short-term changes in ventricular electrophysiological parameters remains insufficiently characterized.

Iron has an essential role in mitochondrial oxidative phosphorylation, cellular energy production, calcium handling, and ion-channel function [8,9]. Accordingly, ID may contribute to alterations in myocardial depolarization and repolarization through impaired cellular energetics and disturbed ionic homeostasis [8,9]. These mechanisms provide a biological rationale for evaluating ECG changes after iron repletion. Nevertheless, surface electrocardiographic (ECG) parameters are indirect surrogate measures of cardiac electrical properties and do not directly demonstrate the occurrence or prevention of clinically significant arrhythmias [10].

The index of cardiac electrophysiological balance (iCEB), defined as the ratio of the QT interval to QRS duration, has been proposed as an ECG-based surrogate of cardiac wavelength that integrates repolarization and conduction properties [11,12,13]. Its heart rate–corrected form, iCEBc, is calculated as the corrected QT interval (QTc) divided by QRS duration. Both increased and decreased iCEB values have been associated with susceptibility to different forms of ventricular arrhythmia: relatively increased values have been linked to torsades de pointes, whereas relatively decreased values may be associated with non-torsades-related ventricular tachyarrhythmias [13,14]. Therefore, a decrease in iCEB or iCEBc should not be considered universally favorable and should be interpreted in relation to baseline values, changes in its QT/QTc and QRS components, and clinical arrhythmic outcomes.

A previous study by Yesil et al. reported changes in the Tpeak-to-Tend (Tp-e) interval and Tp-e/QT and Tp-e/QTc ratios after FCM treatment in patients with heart failure and ID [15]. However, data regarding short-term changes in iCEBc and a broader spectrum of depolarization–repolarization parameters after FCM administration remain limited. Accordingly, the present study aimed to evaluate within-patient changes in iCEBc and other ECG-derived parameters between baseline and 30 ± 5 days after intravenous FCM administration in patients with HFrEF and ID. The primary outcome was the change in iCEBc, whereas changes in a family of 10 secondary ventricular ECG outcomes, including conventional and QRS-adjusted repolarization parameters, were also evaluated. Exploratory analyses examined baseline dependency, the distribution of ΔiCEBc across quartiles of ferritin change, the association between the change in transferrin saturation (TSAT) and ΔiCEBc, and differences according to history of paroxysmal atrial fibrillation. Given the retrospective, uncontrolled design, the study was intended to identify associations and generate hypotheses rather than to establish a causal or direct antiarrhythmic effect.

## 2. Materials and Methods

### 2.1. Study Design and Patient Selection

This retrospective, single-center, paired pre–post study was conducted at a tertiary cardiology outpatient clinic. Adult patients aged ≥ 18 years with clinically stable HFrEF who received intravenous FCM between January 2024 and December 2024 were assessed for eligibility. HFrEF was defined as a left ventricular ejection fraction (LVEF) ≤ 40% on transthoracic echocardiography. ID was defined as a serum ferritin level < 100 ng/mL or a serum ferritin level of 100–299 ng/mL in the presence of TSAT <20% [7].

**Figure 1:**
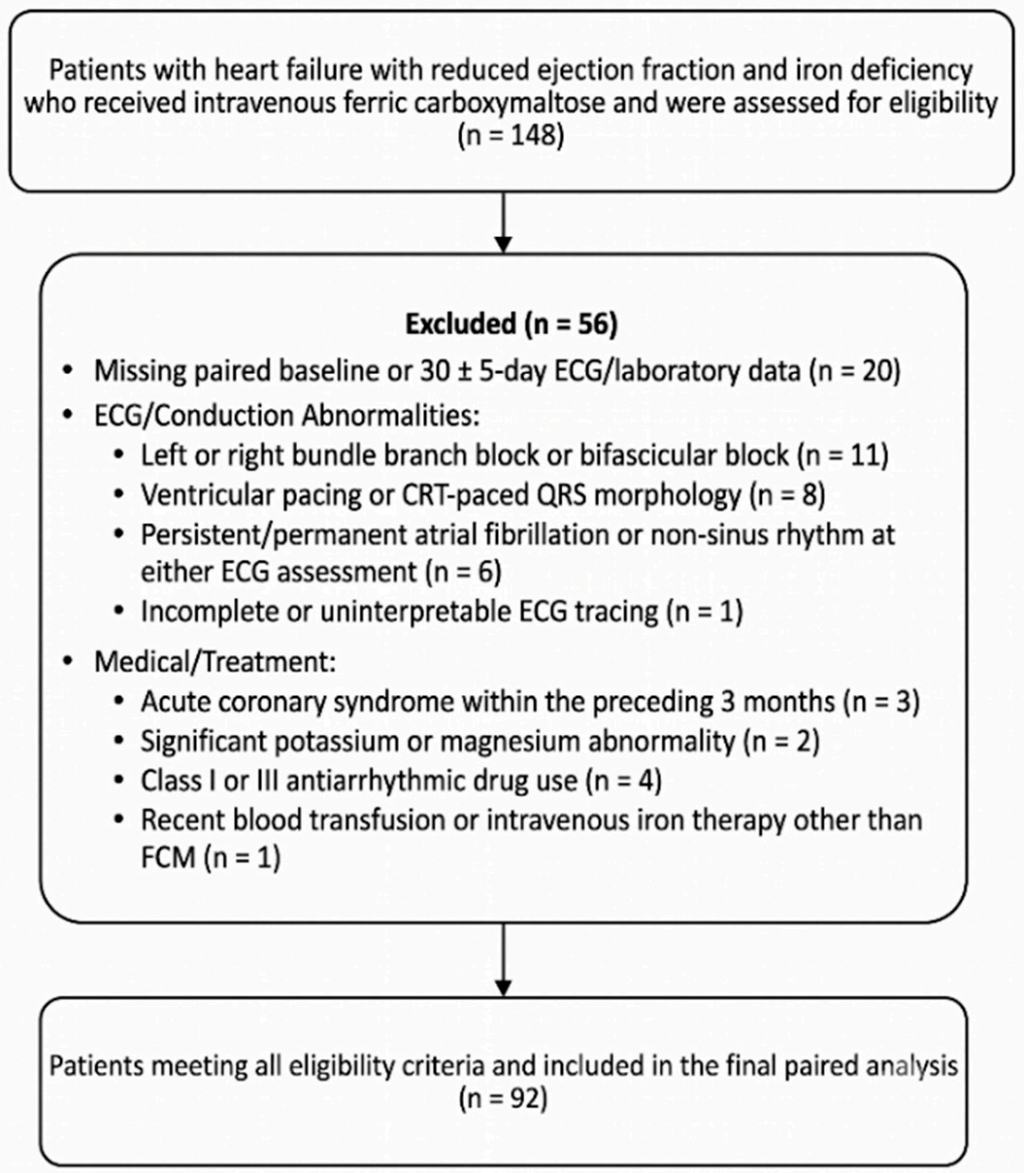
Flow diagram of patient selection. A total of 148 patients with heart failure with reduced ejection fraction and iron deficiency who received intravenous ferric carboxymaltose were assessed for eligibility. Fifty-six patients were excluded according to the predefined eligibility criteria, and 92 patients were included in the final paired analysis. CRT, cardiac resynchronization therapy; ECG, electrocardiogram; FCM, ferric carboxymaltose; HFrEF, heart failure with reduced ejection fraction.

A total of 148 patients with HFrEF and ID who received intravenous FCM during the study period were assessed for eligibility. Of these, 56 patients were excluded: 20 because paired baseline and follow-up ECG or laboratory data were unavailable; 11 because of left or right bundle branch block or bifascicular block; 8 because of ventricular pacing or cardiac resynchronization therapy (CRT)-paced QRS morphology; 6 because of persistent or permanent atrial fibrillation or non-sinus rhythm at either ECG assessment; 4 because of class I or III antiarrhythmic drug use; 3 because of acute coronary syndrome within the preceding 3 months; 2 because of significant potassium or magnesium abnormalities; 1 because of recent blood transfusion or intravenous iron therapy other than FCM; and 1 because of an incomplete or uninterpretable ECG tracing. The remaining 92 patients met all eligibility criteria and were included in the final paired analysis. The patient-selection process is summarized in Figure 1.

The 92 patients analysed here are the same 92 patients reported in the originally submitted manuscript. The eligibility criteria have not been changed and no patient has been reclassified; the six patients in persistent or permanent atrial fibrillation were outside the analysed set in the original submission as well, and are now itemised explicitly in the flow diagram rather than left implicit.

All included patients had standard 12-lead resting ECGs and laboratory measurements available at baseline, before FCM administration, and at 30 ± 5 days after treatment. The protocol-defined 30 ± 5-day interval was selected on both physiological and operational grounds. Serum ferritin and transferrin saturation rise steeply in the first days after a 1000 mg FCM course and reach a plateau at approximately 2–4 weeks, by which time the early erythropoietic response is also largely complete; ECG reassessment at 30 days therefore coincides with the period of maximal biochemical iron repletion rather than with a transient post-infusion peak. The same interval corresponded to the routine post-infusion follow-up visit at our institution, which allowed uniformly timed paired sampling to be obtained retrospectively. The window was chosen to capture early within-patient electrocardiographic changes following completion of FCM administration; it was not intended to assess long-term treatment efficacy or the durability of the observed changes.

Documented HFrEF pharmacotherapy, concomitant medication doses, and device therapy remained stable between the baseline and follow-up assessments. No initiation, discontinuation, or dose adjustment of beta-blockers, renin–angiotensin system inhibitors, mineralocorticoid receptor antagonists (MRAs), sodium–glucose cotransporter 2 (SGLT2) inhibitors, diuretics, or electrolyte supplementation was documented during the study interval. Because medication adherence was not prospectively or independently assessed, treatment stability was defined according to the absence of documented medication or dose changes in the medical records. The duration of guideline-directed medical therapy before the baseline assessment was not systematically recorded and could not be included in the analysis.

Medical records were reviewed for intercurrent clinical events occurring between the two assessments. Patients with a documented acute coronary syndrome, a hospitalization for worsening heart failure, or an acute intercurrent illness during the interval were not identified in the analysed cohort.

The study used a paired pre–post design, with each patient serving as their own control. A concurrent untreated control group was not assembled. Withholding guideline-recommended intravenous iron from an eligible comparator group would not have been clinically or ethically acceptable, and no contemporaneous cohort of untreated iron-deficient patients with HFrEF undergoing paired ECG assessment at a comparable interval was available in the institutional records. The consequences of this design choice for causal interpretation are addressed in the Discussion and Study Limitations Sections.

### 2.2. Non-Inclusion and Exclusion Criteria

Patients were not included if they had acute coronary syndrome within the preceding 3 months; persistent or permanent atrial fibrillation; non-sinus rhythm at either ECG assessment; use of class I or III antiarrhythmic drugs, including amiodarone, sotalol, or flecainide; significant electrolyte abnormalities, defined as serum potassium < 3.5 or >5.5 mmol/L or serum magnesium < 1.6 or >2.4 mg/dL; blood transfusion or intravenous iron therapy other than FCM within the preceding 3 months; missing paired ECG or laboratory data; or incomplete or uninterpretable ECG tracings.

Patients with intraventricular conduction abnormalities, including left or right bundle branch block, bifascicular block, or paced QRS morphology due to a permanent pacemaker or CRT, were also not included, because of the potential influence of these conditions on QRS duration and derived depolarization–repolarization indices. Patients with an implantable cardioverter–defibrillator (ICD) were eligible provided that both ECG recordings showed intrinsic, non-paced QRS complexes.

### 2.3. Ferric Carboxymaltose Administration

All patients received a fixed total dose of 1000 mg of ferric carboxymaltose (Ferinject^®^), administered as two 500 mg intravenous infusions in the outpatient setting. No patient-specific dose adjustment according to body weight, baseline hemoglobin, ferritin, or TSAT was applied. Each infusion was administered over 15–20 min. No serious infusion-related adverse reactions were documented. Because the administered dose was uniform, the achieved degree of iron repletion necessarily varied with body size and with the magnitude of the baseline deficit; this source of heterogeneity is addressed in the Study Limitations Section.

### 2.4. Electrocardiographic Evaluation

Standard 12-lead resting ECGs were recorded using a GE MAC 2000 (GE Healthcare, Milwaukee, WI, USA) electrocardiograph at a paper speed of 25 mm/s and a voltage calibration of 10 mm/mV. ECGs were obtained in the supine position at rest and under stable heart-rate conditions. The time of day at which ECGs were recorded was not standardized and was not retrievable for all tracings.

Two cardiologists, blinded to the patients’ clinical data, independently performed manual measurements on high-resolution digital ECG images using electronic calipers. Automatically generated measurements from the ECG device were not used for any analysed parameter. This applies to the frontal QRS and T-wave axes as well: both were determined manually from the limb-lead net QRS and T-wave deflections by the same two readers, and the values printed by the recorder were not used. For each parameter, the mean of three consecutive sinus cardiac cycles was calculated. When the interobserver difference for an interval measurement exceeded 5 ms, the measurement was jointly re-evaluated and a consensus value was recorded. Formal interobserver and intraobserver reproducibility coefficients were not calculated.

All baseline and follow-up 12-lead ECG tracings were reviewed to confirm the underlying rhythm. All 184 tracings included in the analysis, comprising 92 baseline and 92 follow-up recordings, were obtained during sinus rhythm. Twenty-nine patients (31.5%) had a documented history of paroxysmal atrial fibrillation based on previous electrocardiographic or ambulatory monitoring records; however, none were in atrial fibrillation at the time of either study ECG. Accordingly, all QT and RR interval measurements and all P-wave-based measurements were performed during sinus rhythm.

The QT interval was measured from the onset of the QRS complex to the end of the T wave in the lead with the clearest T-wave termination, preferably lead II or V5. The QTc interval was calculated consistently for all ECG recordings using the Fridericia formula: QTc = QT/RR^1/3^, with QT and RR expressed in seconds. The Fridericia correction was selected in preference to the Bazett formula because Bazett’s square-root correction systematically overcorrects at higher heart rates and undercorrects at lower rates. This limitation is of particular relevance in a heart failure cohort with a mean resting heart rate of 76 bpm, in which Bazett-corrected values would carry a substantial residual dependence on the RR interval. Fridericia’s cube-root correction shows the least residual QT–RR dependence outside the 60–70 bpm range and is the correction recommended for regulatory QT assessment. QRS duration was measured from the onset to the end of the QRS complex in the lead showing the greatest QRS width.

The iCEB was calculated as the QT interval divided by QRS duration:

iCEB = QT/QRS.

The iCEBc was calculated as

iCEBc = QTc/QRS.

The JT and corrected JT (JTc) intervals were calculated patient by patient as QT − QRS and QTc − QRS, respectively, to reduce the contribution of ventricular depolarization duration to the measured repolarization intervals.

The Tp-e interval was measured from the peak to the end of the T wave, typically in leads V5 or V6. The Tp-e/QT and Tp-e/QTc ratios were subsequently calculated. The frontal QRS–T angle was calculated patient by patient as the absolute difference between the frontal-plane QRS axis and T-wave axis. When the absolute difference exceeded 180°, the angle was transformed as 360° minus the absolute difference, yielding values within the conventional 0–180° range.

R-wave peak time (RWPT), representing intraventricular conduction delay, was measured from the onset of the QRS complex to the peak of the R wave in leads III, V1 and V4–V6. Maximum S-wave amplitude, minimum S-wave amplitude and maximum R+S amplitude were also recorded. Maximum P-wave duration (Pmax) and minimum P-wave duration (Pmin) were measured across the 12 ECG leads. P-wave dispersion (PWD) was calculated as the difference between Pmax and Pmin [16]. P-wave measurements were available for the entire study cohort.

### 2.5. Study Outcomes

The primary electrophysiological outcome was the change in iCEBc, calculated as

ΔiCEBc = post-treatment iCEBc − baseline iCEBc.

Negative ΔiCEBc values indicated a numerical reduction in iCEBc, whereas positive values indicated an increase. Because both relatively high and relatively low iCEB values have been associated with different electrophysiological conditions, a reduction in iCEBc was not regarded as universally favorable in isolation.

Secondary ventricular outcomes included changes in QTc, JTc, JT, Tp-e, Tp-e/QT, Tp-e/QTc, frontal QRS–T angle, QT, iCEB, and QRS duration. Atrial, chronotropic and amplitude- or conduction-time-based ECG parameters were considered exploratory outcomes, whereas laboratory parameters were considered supportive outcomes.

Continuous rhythm monitoring and Holter recordings were not performed as part of routine care during the study period, and systematic device interrogation data were not collected for the study interval; stored ICD diagnostics were therefore not available for retrospective retrieval within the study window. Consequently, ventricular arrhythmias, appropriate ICD therapies, sudden cardiac death, and device-detected arrhythmic burden were not evaluated as outcomes.

### 2.6. Statistical Analysis

Continuous variables were assessed for distributional characteristics using visual inspection of histograms and the Shapiro–Wilk test. Normally distributed variables were presented as mean ± standard deviation and non-normally distributed variables as median (interquartile range, Q1–Q3). Categorical variables were presented as counts and percentages. Baseline and follow-up measurements were compared using the paired-samples *t*-test or the Wilcoxon signed-rank test as appropriate. Paired change was defined as the follow-up value minus the baseline value; effect estimates were reported as mean paired differences with 95% confidence intervals (CIs) for normally distributed variables and as Hodges–Lehmann location shifts with 95% CIs otherwise.

The originally specified primary outcome was the within-patient change in iCEBc and was evaluated without multiplicity adjustment. A family of 10 secondary ventricular outcomes—QTc, JTc, JT, Tp-e, Tp-e/QT, Tp-e/QTc, frontal QRS–T angle, QT, iCEB, and QRS duration—was evaluated using the Holm–Bonferroni sequential step-down procedure based on exact unrounded *p*-values. Patients were categorized into quartiles according to the change in serum ferritin, and ΔiCEBc distributions across quartiles were compared using the Kruskal–Wallis test. The relationship between Δferritin and ΔiCEBc was inspected visually using locally weighted scatterplot smoothing. The associations of the change in ferritin and the change in TSAT with ΔiCEBc, and of the changes in TSAT and hemoglobin with ΔQTc and ΔiCEBc, were examined using Spearman rank correlation coefficients. These correlations were exploratory and were reported with nominal, unadjusted *p*-values. These analyses were exploratory and were reported with nominal *p*-values. Atrial, chronotropic and other exploratory ECG outcomes, and laboratory outcomes, were likewise reported with nominal *p*-values.

To evaluate multivariable associations while avoiding the mathematical coupling inherent to change scores, a linear regression model was constructed with follow-up iCEBc as the dependent variable and baseline iCEBc, age, sex, β-blocker use, calcium-channel blocker use, and history of paroxysmal atrial fibrillation as covariates. Variance inflation factors (VIFs) were inspected to assess multicollinearity. Patients were stratified by history of paroxysmal atrial fibrillation in a post-hoc sensitivity analysis. Between-group comparisons of baseline values and paired changes were performed using the independent-samples *t*-test or Mann–Whitney U test, as appropriate.

No a priori sample-size calculation was performed because of the retrospective design; all consecutive patients meeting the eligibility criteria during the study period were included. Observed-power calculations were not performed, because post-hoc power is a deterministic function of the observed *p*-value and adds no information beyond the reported confidence intervals. Instead, the precision achieved is stated directly: with 92 paired observations and the observed within-patient standard deviation of the change in iCEBc of 1.16 units, the study had 80% power to detect a mean paired difference of approximately 0.34 units at a two-sided α of 0.05. Differences smaller than this, and any between-subgroup difference in moderate size, may therefore have escaped detection, and the width of each reported confidence interval should be used to judge the precision of the corresponding estimate.

All tests were two-sided, with *p* < 0.05 considered statistically significant for the primary outcome and after the applicable multiplicity adjustment for secondary outcomes. Analyses were performed using IBM SPSS Statistics version 27.0.1 (IBM Corp., Armonk, NY, USA); figures were generated using Python version 3.11 (Python Software Foundation, Wilmington, DE, USA) and Python-based visualization libraries.

### 2.7. Ethics Approval

The study was conducted in accordance with the Declaration of Helsinki and was approved by the Ankara Etlik City Hospital Scientific Research Evaluation and Ethics Committee (approval number: AEŞH-BADEK2-2025-126; approval date: 20 May 2025). Because of the retrospective design, the requirement for written informed consent was waived.

## 3. Results

### 3.1. Patient Selection and Baseline Characteristics

A total of 148 patients with HFrEF and ID who received intravenous FCM during the study period were assessed for eligibility. Fifty-six patients were excluded according to the predefined eligibility criteria, and 92 patients were included in the final paired analysis (Figure 1). Baseline clinical and pharmacological characteristics are summarized in Table 1.

The cohort had marked iron deficiency, with a median baseline ferritin of 36 (24–78) ng/mL and a mean baseline TSAT of 12.94 ± 2.80%. Absolute iron deficiency was present in 69 patients (75.0%) and functional iron deficiency in 23 (25.0%), so the cohort was dominated by absolute iron deficiency. The cohort was also anemic at baseline: 88 patients (95.7%) met World Health Organization criteria for anemia, and the mean baseline hemoglobin of 9.29 ± 0.95 g/dL lies more than three standard deviations below the diagnostic thresholds for anemia in both sexes.

### 3.2. Changes in Electrocardiographic and Laboratory Parameters

Serum creatinine, potassium, calcium, and magnesium concentrations did not change significantly between the two assessments (Table 2A). Heart rate, PR interval, RR interval, and QRS duration also remained statistically unchanged.

Iron-related laboratory parameters increased during follow-up. Ferritin increased from 36 (24–78) to 362 (190–480) ng/mL, TSAT from 12.94 ± 2.80% to 44.55 ± 7.82%, and hemoglobin from 9.29 ± 0.95 to 11.01 ± 1.01 g/dL (all nominal *p* < 0.001). The mean paired increase in hemoglobin was 1.72 g/dL (95% CI, 1.44 to 2.00).

The originally specified primary outcome, iCEBc, decreased from 4.33 ± 1.04 at baseline to 4.04 ± 0.82 at follow-up, corresponding to a mean paired change of −0.29 (95% CI, −0.53 to −0.05; *p* = 0.016).

Within the family of secondary ventricular outcomes, QTc, JTc, JT, Tp-e, Tp-e/QT, Tp-e/QTc, frontal QRS–T angle, and QT decreased and remained statistically significant after Holm correction (Table 2B). In contrast, the numerical reduction in iCEB did not remain statistically significant after multiplicity adjustment (raw *p* = 0.033; Holm-adjusted *p* = 0.066). QRS duration did not change significantly (mean paired change, +3.40 ms; 95% CI, −3.30 to +10.10; raw and Holm-adjusted *p* = 0.318).

Among the exploratory atrial parameters, Pmax decreased from 113.2 ± 19.8 ms to 103.7 ± 25.3 ms in an unadjusted analysis (mean paired change, −9.50 ms; 95% CI, −14.01 to −5.18; nominal *p* < 0.001), whereas Pmin and PWD did not change significantly (nominal *p* = 0.538 and *p* = 0.110, respectively). Among the exploratory depolarization parameters, RWPT measured in leads III, V1 and V4–V6, maximum and minimum S-wave amplitudes, and maximum R+S amplitude showed no significant change between the two assessments (all nominal *p* > 0.05; Table 2B).

### 3.3. Exploratory Association Between Changes in Iron Indices and iCEBc

Visual inspection using locally weighted scatterplot smoothing did not suggest a clear nonlinear relationship between Δferritin and ΔiCEBc. To explore possible threshold-dependent effects of iron repletion, patients were additionally stratified into quartiles of ferritin change. The distribution of ΔiCEBc did not differ significantly across these quartiles (Kruskal–Wallis *p* = 0.490), with 23 patients included in each quartile. Although numerical differences were observed across quartiles, no statistically supported monotonic or threshold-dependent association was demonstrated, and with 23 patients per quartile the analysis may have been underpowered to detect a modest association.

Spearman rank correlation was then used to examine whether the individual changes in circulating iron and hematologic indices tracked the individual electrocardiographic changes. Transferrin saturation, which reflects circulating iron availability more directly than ferritin does, did not: ΔTSAT was not significantly correlated with ΔiCEBc (ρ = −0.12; *p* = 0.254) or with ΔQTc (ρ = −0.15; *p* = 0.152), and Δferritin was likewise not significantly correlated with ΔiCEBc. The change in hemoglobin behaved differently. ΔHb was inversely correlated with ΔQTc (ρ = −0.21; *p* = 0.044), indicating that patients with a larger rise in hemoglobin showed a greater shortening of QTc, while its correlation with ΔiCEBc did not reach significance (ρ = −0.18; *p* = 0.086). All four coefficients are exploratory and nominal; no multiplicity adjustment was applied to them, and the hemoglobin–QTc coefficient is of small magnitude and would not survive adjustment for the four comparisons reported here. Therefore, within this cohort and over this interval, the magnitude of the individual change in circulating iron indices did not parallel with the magnitude of the individual electrocardiographic change, whereas the magnitude of the correction of anemia showed a weak association with the shortening of QTc.

### 3.4. Individual Variability in ECG-Derived Parameters

**Figure 2:**
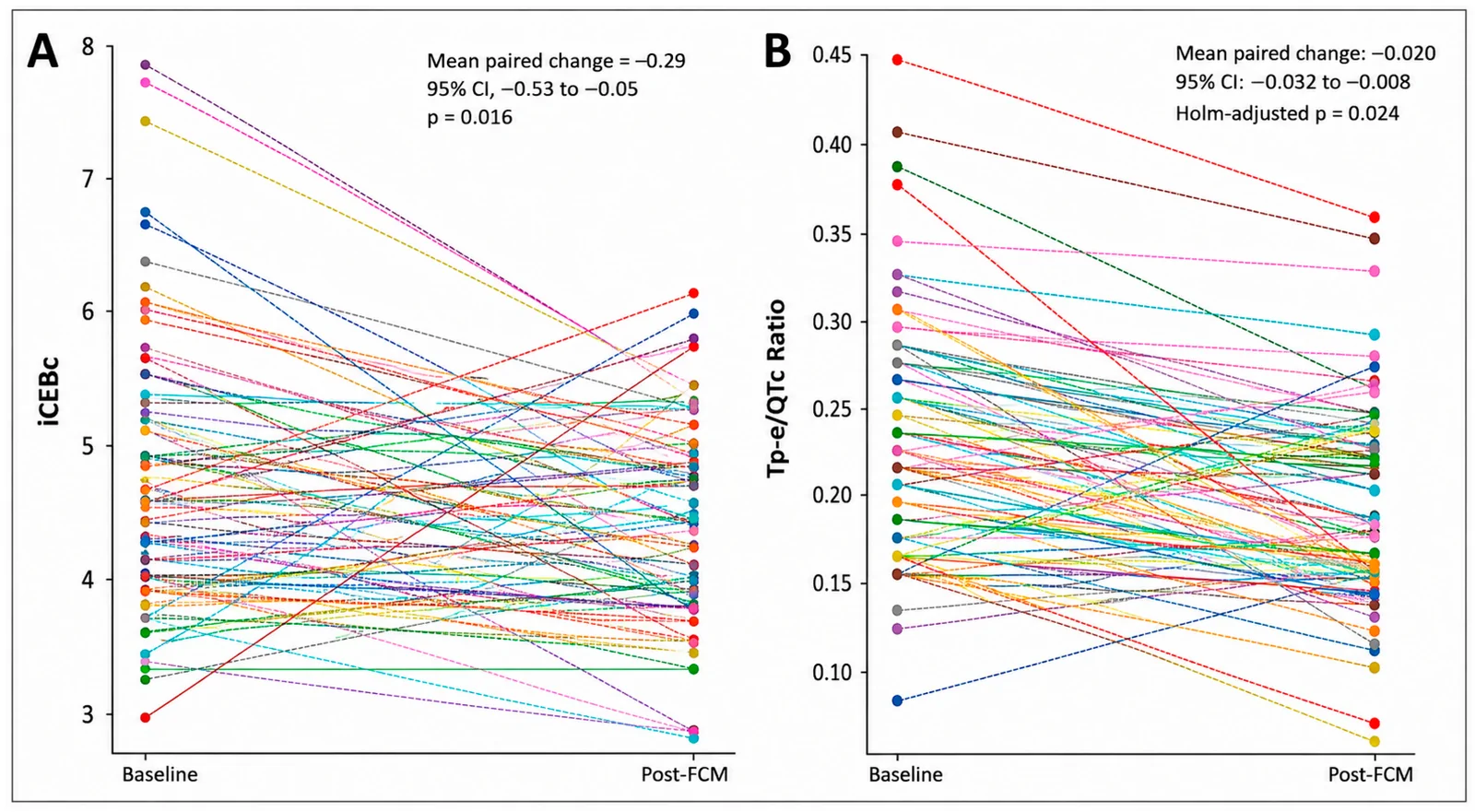
Individual paired changes in selected electrocardiographic parameters following ferric carboxymaltose administration. Individual values obtained at baseline and at 30 ± 5 days after ferric carboxymaltose administration are shown for (**A**) the corrected index of cardiac electrophysiological balance (iCEBc) and (**B**) the Tpeak-to-Tend/QTc ratio (Tp-e/QTc). Each line represents one patient (*n* = 92). iCEBc decreased from 4.33 ± 1.04 to 4.04 ± 0.82, with a mean paired change of −0.29 (95% CI, −0.53 to −0.05; *p* = 0.016). Tp-e/QTc decreased from 0.23 ± 0.06 to 0.21 ± 0.05, with a mean paired change of −0.020 (95% CI, −0.032 to −0.008; raw *p* = 0.004; Holm-adjusted *p* = 0.024). The iCEBc *p*-value corresponds to the originally specified primary outcome and was not adjusted for multiplicity, whereas Tp-e/QTc was included in the multiplicity-adjusted secondary ventricular outcome family. The plots illustrate patient-level variability in the direction and magnitude of change.

Individual paired changes in iCEBc and Tp-e/QTc are presented in Figure 2. Despite the statistically significant cohort-level reductions, considerable between-patient variability was observed, with both numerical decreases and increases occurring at the individual level.

Because no clinically validated directional threshold for ΔiCEBc exists in patients with HFrEF, individual changes in iCEBc were not classified as evidence of clinical benefit or harm.

### 3.5. Baseline-Adjusted Multivariable Analysis

In the exploratory multivariable linear regression model with follow-up iCEBc as the dependent variable (Table 3), baseline iCEBc was the only variable significantly associated with follow-up iCEBc (unstandardized B = 0.52; 95% CI, 0.38 to 0.66; *p* < 0.001). Age, sex, β-blocker use, calcium-channel blocker use, and history of paroxysmal atrial fibrillation were not significantly associated with follow-up iCEBc.

The regression coefficient for baseline iCEBc was below unity and was compatible with partial convergence of follow-up values toward the cohort mean. Because ΔiCEBc mathematically incorporates the baseline value, unadjusted associations between baseline iCEBc and subsequent change are susceptible to mathematical coupling and regression to the mean. Accordingly, baseline-dependent change-score interpretations were considered exploratory and were not used to identify patients with a clinically distinct response to FCM administration.

Mineralocorticoid receptor antagonist use and sodium–glucose cotransporter 2 inhibitor use were not entered into the model. With 92 observations, six covariates and an SGLT2 inhibitor prevalence of 14.1%, the model could not accommodate further terms without exceeding the observations-per-covariate ratio conventionally regarded as the lower bound for stable linear-regression estimation, and coefficients derived from such a model would not have been interpretable. This constraint, and the residual confounding from background pharmacotherapy that follows from it, are stated in the Study Limitations Section.

### 3.6. Post-Hoc Analysis Stratified by History of Paroxysmal Atrial Fibrillation

In the post-hoc analysis stratified by history of paroxysmal atrial fibrillation (Supplementary Table S1), patients with a history of paroxysmal atrial fibrillation had higher baseline QTc (466.2 ± 32.5 vs. 449.0 ± 28.1 ms; nominal *p* = 0.012), Pmax (121.4 ± 20.8 vs. 109.4 ± 18.2 ms; nominal *p* = 0.006), and PWD [68 (48–86) vs. 56 (40–72) ms; nominal *p* = 0.018] than patients without such a history.

No statistically significant between-group differences were observed in changes in iCEBc, QTc, JTc, Pmax, or PWD. However, the subgroup sizes were limited to 29 patients with and 63 patients without a history of paroxysmal atrial fibrillation. Therefore, this post-hoc analysis had limited power to exclude potentially meaningful differences between the groups.

## 4. Discussion

In this retrospective paired pre–post study of patients with HFrEF and ID, intravenous FCM administration was associated with short-term reductions in several electrocardiographic indices of ventricular repolarization. The originally specified primary outcome, iCEBc, decreased significantly over the 30 ± 5-day assessment interval. QTc, JTc, JT, Tp-e, Tp-e/QT, Tp-e/QTc, frontal QRS–T angle, and QT also decreased and remained statistically significant after Holm correction, whereas QRS duration did not change significantly. In contrast, the reduction in iCEB did not remain statistically significant after multiplicity adjustment. These findings should be interpreted as short-term changes in ECG-derived surrogate parameters rather than as evidence of a causal or direct antiarrhythmic effect.

Several observations temper the interpretation of iCEBc in this cohort. Although the originally specified primary outcome decreased significantly, the statistical evidence for this change was less robust than that observed for QTc, JTc, JT, and Tp-e. Because iCEBc is a ratio in which QRS duration forms the denominator, and because QRS duration did not change significantly, the observed reduction in iCEBc appears to have been driven predominantly by shortening of its QTc numerator. The patient-level JT and JTc analyses, which subtract QRS duration rather than incorporating it as a denominator, showed changes in the same direction and with greater statistical strength. Collectively, these findings support an interpretation based primarily on repolarization shortening rather than on a change in depolarization–repolarization balance as such. On the available evidence, iCEBc did not outperform conventional repolarization indices in this setting, and no claim of incremental sensitivity can be made.

A numerical reduction in iCEBc cannot be interpreted as uniformly favorable. Experimental and clinical observations suggest that both relatively elevated and relatively reduced iCEB values may be associated with arrhythmic vulnerability. Increased values have been linked to torsades de pointes in the setting of prolonged repolarization, whereas reduced values may accompany non-torsades ventricular tachyarrhythmias in the setting of relative repolarization shortening [13,14]. Reference values for iCEB have been derived mainly from healthy volunteers and from drug-safety cohorts, and no validated reference range or directional threshold has been established for iCEBc in patients with HFrEF, in whom baseline QRS duration and repolarization are already abnormal. For this reason the values observed here cannot be compared against a normal range, and the direction of change cannot be classified as favorable or unfavorable at the individual level. The heterogeneity of individual trajectories in the present cohort, with numerical decreases in some patients and increases in others, further emphasizes the need for bidirectional interpretation. We therefore describe the findings as numerical changes in an ECG-derived surrogate parameter and do not characterize a reduction in iCEBc as electrophysiological improvement. In particular, a further reduction in patients whose baseline values are already relatively low would not necessarily be desirable.

The direction and magnitude of the individual trajectories varied considerably, and several sources of this heterogeneity can be identified even though none can be quantified in the present dataset. First, each patient contributes a single 10 s tracing at each time point, so that spontaneous biological fluctuation in repolarization and the measurement error inherent to manual interval assessment are both incorporated undiluted into every individual change score; with a within-patient standard deviation of the change in iCEBc of 1.16 units against a mean change of −0.29, measurement and biological noise alone are sufficient to produce changes in either direction at the individual level. Second, a uniform 1000 mg dose was given irrespective of body size and of the magnitude of the baseline deficit, so the proportional correction of the iron deficit—and, correspondingly, the rise in hemoglobin—differed substantially between patients. Third, patients differed in baseline repolarization reserve: those with the most prolonged baseline QTc had the greatest scope for shortening, whereas patients already near the lower end of the observed distribution had little. Fourth, autonomic tone, volume status and the time of day of recording were neither standardized nor retrievable, and each influences ventricular repolarization independently of iron status. The aggregate consequence is that the cohort-level mean change is interpretable whereas an individual patient’s change is not, which is the principal reason that no responder classification is reported.

An important alternative explanation must be considered for the observed repolarization changes. The mean baseline hemoglobin in this cohort was 9.29 ± 0.95 g/dL, so that essentially all patients were anemic at study entry, and hemoglobin rose by a mean of 1.72 g/dL over the assessment interval. Anemia is itself associated with prolongation of ventricular repolarization and with increased transmural dispersion, and correction of anemia has been reported to shorten QTc and Tp-e in other clinical settings. Because iron repletion and correction of anemia occurred together and could not be separated in this design, the observed reductions in QTc, JT, JTc and Tp-e may reflect the hemodynamic and rheological consequences of a rising hemoglobin concentration rather than a direct electrophysiological effect of restored myocardial iron availability. The exploratory correlation analyses give this alternative some direct support and we consider it important to say so: the change in hemoglobin was inversely correlated with the change in QTc (ρ = −0.21; *p* = 0.044), whereas neither circulating iron index was correlated with any electrocardiographic change. The coefficient is small, it is nominal and unadjusted, and it would not survive correction for the four exploratory correlations reported, so it should not be treated as evidence that correction of anemia is the operative mechanism. It does, however, mean that the anemia hypothesis is the only one of the two for which any supporting signal was observed in these data, and it should be weighed accordingly. This cohort was also substantially more anemic than the populations enrolled in the pivotal intravenous iron trials, which limits extrapolation of the present observations to non-anemic iron-deficient patients with HFrEF, in whom the same ECG changes would not necessarily be expected. Disentangling the two mechanisms would require a comparison of anemic and non-anemic iron-deficient patients, or a design in which the change in hemoglobin varies independently of the change in iron status; neither was available here, since 88 of the 92 patients (95.7%) were anemic at baseline.

The present findings are directionally concordant with those reported by Yesil et al., who observed reductions in Tp-e, Tp-e/QT, and Tp-e/QTc after FCM administration in patients with heart failure and ID [15]. The current study extends that work by evaluating the depolarization–repolarization ratio indices iCEB and iCEBc and the QRS-excluded JT and JTc intervals, which were not previously reported in this clinical context. In addition, multiplicity was formally addressed across a family of secondary ventricular outcomes, and baseline dependency was examined using a baseline-adjusted regression model. However, the present study does not extend the previous evidence in the clinically most important direction, because ventricular arrhythmias, device therapies, and other arrhythmic outcomes were not assessed.

The biological relationship between ID and myocardial electrical properties is likely multifactorial. Iron participates in mitochondrial oxidative phosphorylation, cellular energy production, calcium handling, and several ion-channel-dependent processes [8,9,17,18]. ID may therefore contribute to altered myocardial energetics and ionic homeostasis, potentially influencing ventricular repolarization. Restoration of iron availability could plausibly modify these processes. Nevertheless, the present study did not measure myocardial iron content, intracellular energetics, ion-channel function, or excitation–contraction coupling. Accordingly, these mechanisms provide biological plausibility but cannot establish the mechanism responsible for the observed ECG changes.

In the exploratory analyses, neither the change in ferritin nor the change in transferrin saturation was significantly correlated with the change in iCEBc, ΔTSAT was likewise uncorrelated with ΔQTc, and ΔiCEBc did not differ significantly across quartiles of ferritin change. Transferrin saturation was examined specifically because it reflects circulating iron availability more directly than ferritin does, so the absence of an association for both indices is more informative than the ferritin result alone would have been. Three readings of this pattern are compatible with our data and we cannot distinguish between them. The first is biological: circulating iron indices describe the transport and storage compartments, whereas any electrophysiological effect of iron repletion would be mediated at the level of cardiomyocyte mitochondrial function and calcium handling, and these compartments need not equilibrate on the same timescale, so the magnitude of the rise in a circulating index need not predict the magnitude of any tissue-level change. The second is statistical: individual change scores in both the exposure and the outcome carry substantial measurement and biological noise, correlations between two such quantities are attenuated accordingly, and with 92 patients a modest true association would not have been detected. The third is that the electrocardiographic changes are not driven by iron repletion at all. The observation that the change in hemoglobin, unlike either iron index, did show a weak inverse correlation with the change in QTc is consistent with this third reading, although the coefficient is small and unadjusted and cannot carry that argument on its own. We therefore report these associations as observations rather than as evidence of a tissue-level mechanism. Each quartile included only 23 patients, and the analysis may have been underpowered to detect a modest threshold effect. Ferritin is an acute-phase reactant and may be influenced by inflammation and comorbidities common in heart failure [19]. Systemic inflammation is a persistent feature of chronic heart failure and interacts with iron handling through hepcidin-mediated pathways, so that circulating ferritin may rise for reasons unrelated to repletion of tissue iron stores [20]. Changes in circulating ferritin may therefore not fully reflect functional or tissue-level iron availability. Because systematic inflammatory biomarkers and measures of myocardial or intracellular iron were unavailable, these findings should be interpreted cautiously and should not be generalized to other indicators of functional iron availability.

The baseline-adjusted multivariable analysis demonstrated that baseline iCEBc was associated with follow-up iCEBc, whereas age, sex, β-blocker use, calcium-channel blocker use, and history of paroxysmal atrial fibrillation were not significantly associated with the follow-up value. The regression coefficient for baseline iCEBc was below unity, which was compatible with partial convergence of follow-up values toward the cohort mean. Because ΔiCEBc mathematically incorporates baseline iCEBc, the strong unadjusted association between baseline values and subsequent change is susceptible to mathematical coupling and regression to the mean. Consequently, baseline-dependent change-score findings should not be interpreted as showing that patients with higher baseline iCEBc derive greater treatment benefit or are more likely to respond to FCM.

In the post hoc analysis stratified by history of paroxysmal atrial fibrillation, patients with such a history had higher baseline QTc, Pmax, and PWD values. However, no statistically significant between-group differences were observed in changes in iCEBc, QTc, JTc, Pmax, or PWD. Given the limited subgroup sizes, these findings should not be interpreted as evidence that atrial fibrillation history has no influence on the observed changes. Rather, the analysis was insufficiently powered to exclude a clinically meaningful interaction. For the same reason, no formal subgroup analyses were undertaken according to New York Heart Association class, LVEF stratum, heart failure etiology, or ICD status: with 92 patients and a single paired assessment, such analyses would have had very low power and a high probability of spurious findings, and any apparent interaction would not have been interpretable.

The clinical role that iCEBc might eventually occupy in this population cannot be defined from the present data, and we deliberately refrain from proposing one. If a role exists, it would most plausibly be as a summary marker of the combined depolarization–repolarization state that could be tracked serially during iron repletion at negligible cost, in the same way that QTc is monitored during exposure to QT-prolonging drugs. Establishing that role would require, at a minimum, reference ranges derived in HFrEF cohorts, evidence that a change in a given magnitude carries prognostic information independent of QTc and JTc, and demonstration in a controlled design that the marker responds to treatment rather than to time. The present study meets none of these conditions, and until they are met iCEBc should be regarded as a research parameter rather than a clinical tool.

Taken together, the present findings indicate that FCM administration was associated with short-term changes in several ECG-derived repolarization parameters in patients with HFrEF and ID. However, the uncontrolled design, the concurrent correction of anemia, individual heterogeneity, susceptibility of change-score analyses to regression to the mean, and absence of clinical arrhythmic outcomes preclude interpretation of these findings as evidence of electrical stabilization or arrhythmia prevention. Whether the observed changes persist beyond one month is unknown; iron indices commonly decline again within 3–6 months of a single FCM course, so a repeat assessment at that interval would establish whether the ECG changes track iron status or represent a transient post-infusion phenomenon. Prospective controlled studies incorporating serial rhythm monitoring, systematic device interrogation, validated reference ranges for iCEBc, inflammatory and functional iron biomarkers, adjustment for the change in hemoglobin, and follow-up extending to at least 6 and 12 months are required to determine the clinical relevance of these observations.

### Study Limitations

This study has several limitations. First, the retrospective, single-center, single-arm pre–post design lacked a non-treated control group. Although within-patient paired comparisons reduce inter-individual variability, spontaneous temporal variation, changes related to background heart-failure care, residual confounding, and regression to the mean cannot be excluded. Consequently, no causal inference regarding FCM administration can be made.

Second, hemoglobin rose substantially over the assessment interval in a cohort that was anemic at baseline. Correction of anemia is therefore an unmeasured co-intervention that cannot be separated from iron repletion, and it provides an alternative and equally plausible explanation for the observed repolarization changes.

Third, the coefficient for baseline iCEBc in the baseline-adjusted regression model was below unity and was compatible with partial convergence of follow-up values toward the cohort mean. This finding, together with the mathematical dependency between baseline iCEBc and ΔiCEBc, reinforces the exploratory nature of baseline-dependent change-score interpretations. Such findings cannot identify treatment responders or establish that baseline iCEBc predicts benefit from FCM.

Fourth, no clinical arrhythmic outcomes were available. Systematic Holter monitoring and longitudinal device-interrogation data were not collected during the study period. Therefore, ventricular arrhythmias, appropriate ICD therapies, sudden cardiac death, and device-detected arrhythmic burden could not be evaluated. The ECG findings must consequently be regarded as surrogate changes and not as evidence of a direct antiarrhythmic effect.

Fifth, all electrophysiological information was derived from the standard 10 s, 12-lead surface ECG. This modality samples a single brief epoch of cardiac activity, cannot capture beat-to-beat or diurnal variability in repolarization, and does not provide measures such as microvolt T-wave alternans, heart-rate variability, or dispersion mapping. The observed parameters therefore represent a limited window onto ventricular electrophysiology.

Sixth, formal interobserver and intraobserver reproducibility coefficients could not be calculated because separate repeated measurements were not retained in the retrospective dataset. Measurements differing by more than 5 ms between the two blinded readers were jointly re-evaluated and resolved by consensus; however, this approach does not substitute for formal assessment using intraclass correlation coefficients.

Seventh, although the readers were blinded to the patients’ clinical data, baseline and follow-up tracings were assessed as paired examinations, and blinding to their temporal sequence was not feasible. This may have introduced measurement expectation bias.

Eighth, the protocol-defined 30 ± 5-day assessment window captured only early changes following FCM administration. The study therefore cannot determine the durability of the observed findings or their relationship with longer-term clinical outcomes.

Ninth, a fixed total dose of 1000 mg was administered to all patients irrespective of body weight and baseline iron indices. The proportional correction of the iron deficit therefore differed between patients, which may have contributed to the heterogeneity of individual electrophysiological trajectories and precludes any assessment of a dose–response relationship.

Tenth, body mass index, heart-rate variability indices, and the time of day at which ECGs were recorded were not available. Circadian variation in autonomic tone and in ventricular repolarization could therefore not be accounted for, and paired tracings were not necessarily obtained at comparable times of day.

Eleventh, the duration of guideline-directed medical therapy before the baseline ECG was not recorded. Patients in whom therapy had recently been initiated or up-titrated may still have been undergoing reverse remodeling, which could contribute to changes in repolarization independent of iron status.

Twelfth, the multivariable model did not include mineralocorticoid receptor antagonists or sodium–glucose cotransporter 2 inhibitors, both of which have recognized effects on remodeling and on ventricular electrophysiology. The number of paired observations constrained the number of covariates that could be modelled without overfitting, and residual confounding from background pharmacotherapy therefore cannot be excluded.

Thirteenth, patients with bundle branch block, paced QRS complexes, CRT-paced morphology, and major electrolyte abnormalities were not included. Although these criteria improved the interpretability of QRS-dependent ECG indices, they limit the applicability of the findings to the broader HFrEF population, particularly patients with conduction disease or resynchronization devices.

Fourteenth, systematic inflammatory biomarkers and measures of myocardial or intracellular iron content were unavailable. Ferritin may be influenced by inflammation and by comorbidities common in heart failure, limiting its ability to represent functional or tissue-level iron availability [19,20].

Fifteenth, the post-hoc subgroup analysis stratified by history of paroxysmal atrial fibrillation was limited by the relatively small subgroup sizes and was not adequately powered to detect an interaction or differential response.

Sixteenth, paired hematocrit values and reticulocyte indices were not retrievable for the study interval, because they were not part of the routine laboratory panel obtained at the post-infusion visit. The erythropoietic response to FCM could therefore be characterized only through the change in hemoglobin, and the reticulocyte kinetics that would distinguish an early proliferative response from a later steady-state rise could not be examined.

Finally, the study was conducted at a single tertiary center in Türkiye, in a cohort recruited from a single national health system and a single dietary and genetic background, and no formal a priori sample-size calculation was performed. Baseline iron and hemoglobin status in this population is more severe than that reported in the pivotal European intravenous iron trials, and thresholds for referral for intravenous iron, the availability of ferric carboxymaltose, and background heart-failure pharmacotherapy—most notably the low observed uptake of sodium–glucose cotransporter 2 inhibitors—differ from those in other settings. External validity beyond comparably iron-depleted, comparably treated populations should therefore not be assumed. With 92 paired observations the study was able to detect a mean paired change in iCEBc of approximately 0.34 units with 80% power; smaller effects and moderate between-subgroup differences may have gone undetected. The findings should therefore be regarded as hypothesis-generating and require confirmation in adequately powered, prospective, controlled studies with predefined ECG and clinical arrhythmic endpoints.

## 5. Conclusions

In this retrospective single-arm cohort of patients with HFrEF and ID, intravenous FCM administration was associated with short-term reductions in several ECG-derived markers of ventricular repolarization over a 30 ± 5-day interval. The statistical evidence for the change in the originally specified primary outcome, iCEBc, was less robust than that observed for several conventional repolarization intervals, and iCEBc did not demonstrate incremental sensitivity in this setting.

These parameters are surrogate measures, and a numerical reduction in iCEBc cannot be regarded as uniformly favorable. Because hemoglobin rose concurrently in a cohort in which 95.7% of patients were anemic at baseline, correction of anemia cannot be separated from iron repletion as an explanation for the observed changes; indeed, the change in hemoglobin was the only exposure examined that showed any association with the electrocardiographic changes, whereas neither circulating iron index did. The absence of a non-treated control group and of clinical arrhythmic outcomes precludes any causal inference or conclusion regarding an antiarrhythmic effect. These findings are hypothesis-generating and warrant evaluation in prospective controlled studies incorporating rhythm monitoring, systematic device interrogation, and predefined arrhythmic endpoints.

## Figures and Tables

**Table 1:** Baseline clinical, demographic, and pharmacological characteristics of the study cohort.

Variable	Value (*n* = 92)
Age, years	65.5 ± 10.8
Male sex, *n* (%)	55 (59.8)
Smoking, *n* (%)	40 (43.5)
Alcohol use, *n* (%)	9 (9.8)
Hypertension, *n* (%)	29 (31.5)
Diabetes mellitus, *n* (%)	20 (21.7)
Coronary artery disease, *n* (%)	39 (42.4)
History of paroxysmal atrial fibrillation, *n* (%) ^a^	29 (31.5)
Chronic kidney disease, *n* (%)	26 (28.3)
NYHA class II / III, *n* (%)	50 (54.3)/42 (45.7)
LVEF, %	30.6 ± 6.8
LVEDD, mm	60.2 ± 5.7
sPAP, mmHg	42.3 ± 9.5
Anemia (WHO criteria), *n* (%) ^b^	88 (95.7)
Absolute iron deficiency, *n* (%) ^c^	69 (75.0)
Functional iron deficiency, *n* (%) ^c^	23 (25.0)
ACE inhibitor or ARB use, *n* (%)	81 (88.0)
β-blocker use, *n* (%)	86 (93.5)
SGLT2 inhibitor use, *n* (%)	13 (14.1)
MRA use, *n* (%)	52 (56.5)
Calcium-channel blocker use, *n* (%)	11 (12.0)
Loop diuretic use, *n* (%)	76 (82.6)
ICD implantation, *n* (%) ^d^	29 (31.5)
Total FCM dose, mg	1000 in all patients
Protocol-defined follow-up ECG window, days	30 ± 5

^a^ All baseline and follow-up ECGs included in the analysis were recorded during confirmed sinus rhythm; the reported value refers to a documented history of paroxysmal atrial fibrillation. ^b^ Hemoglobin < 13 g/dL in men and <12 g/dL in women. ^c^ Absolute iron deficiency, ferritin < 100 ng/mL; functional iron deficiency, ferritin 100–299 ng/mL with transferrin saturation < 20%. ^d^ All ICD carriers exhibited intrinsic QRS complexes with no evidence of ventricular pacing. Patients with CRT devices or continuous ventricular pacing were not included per the eligibility criteria. Abbreviations: ACE, angiotensin-converting enzyme; ARB, angiotensin receptor blocker; CRT, cardiac resynchronization therapy; ECG, electrocardiogram; FCM, ferric carboxymaltose; ICD, implantable cardioverter–defibrillator; LVEDD, left ventricular end-diastolic diameter; LVEF, left ventricular ejection fraction; MRA, mineralocorticoid receptor antagonist; NYHA, New York Heart Association; sPAP, systolic pulmonary artery pressure; SGLT2, sodium–glucose cotransporter 2; WHO, World Health Organization.

**Table 2:** (**A**). Biochemical and safety parameters before and after ferric carboxymaltose administration. (**B**). Electrocardiographic outcomes before and after ferric carboxymaltose administration.

(**A**)					
**Variable**	**Before**	**After**	**Paired Change (95% CI) ^a^**	***p***	
Hemoglobin, g/dL	9.29 ± 0.95	11.01 ± 1.01	+1.72 (+1.44 to +2.00)	<0.001	
Ferritin, ng/mL ^b^	36 (24–78)	362 (190–480)	+318 (+272 to +365)	<0.001	
Transferrin saturation, %	12.94 ± 2.80	44.55 ± 7.82	+31.61 (+29.99 to +33.23)	<0.001	
Serum creatinine, mg/dL ^b^	1.2 (0.9–1.5)	1.2 (1.0–1.6)	0.00 (−0.05 to +0.05)	0.881	
Serum potassium, mmol/L ^b^	4.3 (4.0–4.6)	4.2 (4.0–4.5)	−0.10 (−0.20 to 0.00)	0.448	
Serum calcium, mg/dL ^b^	9.0 (8.6–9.4)	9.1 (8.6–9.5)	+0.05 (−0.10 to +0.15)	0.647	
Serum magnesium, mg/dL ^b^	2.1 (1.8–2.5)	2.1 (1.9–2.4)	0.00 (−0.08 to +0.08)	0.782	
(**B**)					
**Variable**	**Before**	**After**	**Paired Change (95% CI) ^a^**	**Raw *p***	**Holm-Adjusted *p***
***Primary electrophysiological outcome***					
iCEBc (QTc/QRS) ^b^	4.33 ± 1.04	4.04 ± 0.82	−0.29 (−0.53 to −0.05)	0.016	—
***Secondary ventricular family (m = 10)***					
QTc interval, ms	454.4 ± 30.2	438.3 ± 28.5	−16.10 (−20.81 to −11.22)	<0.001	<0.001
JTc interval (QTc − QRS), ms ^c^	344.2 ± 24.8	324.7 ± 22.1	−19.50 (−24.19 to −14.81)	<0.001	<0.001
JT interval (QT − QRS), ms ^c^	321.2 ± 32.4	304.0 ± 34.2	−17.20 (−25.50 to −8.90)	<0.001	<0.001
Tp-e interval, ms	106.7 ± 27.7	95.5 ± 25.3	−11.20 (−16.21 to −6.07)	<0.001	<0.001
Tp-e/QT ratio	0.25 ± 0.07	0.23 ± 0.07	−0.018 (−0.030 to −0.005)	0.004	0.024
Tp-e/QTc ratio	0.23 ± 0.06	0.21 ± 0.05	−0.020 (−0.032 to −0.008)	0.004	0.024
Frontal QRS–T angle, ° ^d^	78 (42–115)	62 (34–92)	−16.0 (−25.00 to −6.00)	0.005	0.024
QT interval, ms	431.4 ± 40.6	417.6 ± 44.6	−13.80 (−24.15 to −3.52)	0.006	0.024
iCEB (QT/QRS)	4.10 ± 0.98	3.84 ± 0.80	−0.26 (−0.50 to −0.02)	0.033	0.066
QRS duration, ms	110.2 ± 24.7	113.6 ± 28.1	+3.40 (−3.30 to +10.10)	0.318	0.318
***Exploratory atrial and chronotropic parameters*** ^e^					
Heart rate, bpm	76.3 ± 11.0	76.2 ± 9.5	−0.10 (−1.80 to +1.60)	0.849	—
PR interval, ms	171.2 ± 33.3	162.4 ± 39.2	−8.80 (−18.10 to +0.50)	0.064	—
RR interval, ms	806.9 ± 156.8	820.3 ± 152.2	+13.40 (−7.20 to +34.00)	0.201	—
Pmax, ms	113.2 ± 19.8	103.7 ± 25.3	−9.50 (−14.01 to −5.18)	<0.001	—
Pmin, ms	50.0 ± 16.6	48.2 ± 15.7	−1.80 (−7.50 to +3.90)	0.538	—
P-wave dispersion, ms	60 (42–78)	40 (28–58)	−7.70 (−17.20 to +1.80)	0.110	—
***Exploratory depolarization parameters*** ^e,f^					
R-wave peak time, overall, ms	40 (30–50)	40 (30–45)	0.00 (−2.50 to +2.50)	0.065	—
RWPT V1, ms	10 (10–20)	15 (10–20)	0.00 (−2.00 to +2.00)	0.641	—
RWPT V4–V6, ms	20 (20–35)	20 (20–35)	0.00 (−2.50 to +2.50)	0.115	—
RWPT III, ms	20 (15–30)	20 (15–30)	0.00 (−2.00 to +2.00)	0.616	—
Maximum S-wave amplitude, mm	14 (8–22)	14 (7–20)	0.00 (−1.50 to +1.50)	0.519	—
Minimum S-wave amplitude, mm	1 (1–3)	1 (1–3)	0.00 (−0.50 to +0.50)	0.530	—
Maximum R+S amplitude, mm	15 (10–24)	17 (11–25)	+1.00 (−1.50 to +2.50)	0.575	—

(**A**). ^a^ Paired change is follow-up minus baseline: mean paired difference for normally distributed variables, Hodges–Lehmann location shift for non-normally distributed variables. Positive values indicate an increase. ^b^ Presented as median (interquartile range, Q1–Q3); paired comparison by Wilcoxon signed-rank test. Laboratory outcomes were supportive and were not included in the ventricular multiplicity family; *p*-values are unadjusted. (**B**). ^a^ Paired change is follow-up minus baseline; patient-level mean differences or Hodges–Lehmann location shifts as appropriate. ^b^ The originally specified primary outcome was evaluated without multiplicity adjustment. The Holm–Bonferroni step-down procedure was applied across a family of 10 secondary ventricular outcomes using exact unrounded raw *p*-values. After adjustment, the reduction in iCEB was no longer statistically significant. ^c^ JT and JTc were calculated patient by patient as QT − QRS and QTc − QRS to reduce the contribution of depolarization duration to the measured repolarization intervals. ^d^ The frontal QRS–T angle was recalculated patient by patient within the conventional 0–180° range using |QRS axis − T axis|, and 360° − difference where the absolute difference exceeded 180°. ^e^ Exploratory outcomes; *p*-values are nominal and unadjusted. ^f^ Non-normally distributed exploratory depolarization parameters are presented as median (interquartile range, Q1–Q3); paired comparisons were performed using the Wilcoxon signed-rank test, and paired change estimates are reported as Hodges–Lehmann location shifts with 95% confidence intervals. Abbreviations: CI, confidence interval; iCEB, index of cardiac electrophysiological balance; iCEBc, corrected index of cardiac electrophysiological balance; JTc, corrected JT interval; Pmax, maximum P-wave duration; Pmin, minimum P-wave duration; PWD, P-wave dispersion; QRS, QRS complex duration; QTc, corrected QT interval; RWPT, R-wave peak time; Tp-e, Tpeak-to-Tend interval.

**Table 3:** Multivariable linear regression model with follow-up iCEBc as the dependent variable.

Independent Variable	B (95% CI)	Standardized β	*p*	VIF
Baseline iCEBc, per 1 unit	0.52 (0.38 to 0.66)	0.58	<0.001	1.15
Age, per 1 year	0.005 (−0.005 to 0.015)	0.07	0.321	1.08
Male sex	−0.07 (−0.29 to 0.15)	−0.04	0.521	1.11
β-blocker use	−0.12 (−0.48 to 0.24)	−0.05	0.512	1.09
Calcium-channel blocker use	−0.09 (−0.45 to 0.27)	−0.04	0.621	1.06
History of paroxysmal AF	−0.11 (−0.38 to 0.16)	−0.06	0.428	1.14

n = 92; adjusted R^2^ = 0.44; RMSE = 0.59; maximum VIF = 1.15. The model was specified with follow-up iCEBc rather than ΔiCEBc as the dependent variable, because the change score mathematically incorporates the baseline value. A baseline iCEBc coefficient below unity (B = 0.52) was compatible with partial convergence of follow-up values toward the cohort mean. AF, atrial fibrillation; VIF, variance inflation factor.

## Data Availability

The data presented in this study are available on request from the corresponding author.

## Supplementary Materials

The following supporting information can be downloaded at https://www.mdpi.com/article/10.3390/medicina62091700/s1. Table S1. Electrophysiological changes stratified by history of paroxysmal atrial fibrillation.
