# Supplementary material for: Ferric Carboxymaltose Therapy Improves the Index of Cardiac Electrophysiological Balance in Iron-Deficient Heart Failure with Reduced Ejection Fraction
Source: Medicina (Kaunas). 2026 Sep 4;62(9):1700. doi: 10.3390/medicina62091700 (PMC13609117; doi:10.3390/medicina62091700)
Supplement: Supplementary file 1 [file medicina-62-01700-s001.zip › medicina-4398112-supplementary.pdf]

Supplementary Table S1. Electrophysiological changes stratified by history of paroxysmal atrial fibrillation.

**Variable (sinus rhythm tracings)**	**History of paroxysmal AF (n = 29)**	**No history of AF (n = 63)**	**Between-group *p* [a]**
Baseline values			
Baseline iCEBc	4.61 ± 1.12	4.20 ± 0.95	0.081
Baseline QTc, ms	466.2 ± 32.5	449.0 ± 28.1	0.012
Baseline Pmax, ms	121.4 ± 20.8	109.4 ± 18.2	0.006
Baseline PWD, ms	68 (48–86)	56 (40–72)	0.018
Paired changes (Δ = follow-up − baseline), with 95% CI			
ΔiCEBc	−0.38 (−0.72 to −0.04)	−0.25 (−0.52 to +0.02)	0.214
ΔQTc, ms	−18.2 (−25.1 to −11.3)	−15.1 (−20.8 to −9.4)	0.482
ΔJTc, ms	−21.4 (−28.2 to −14.6)	−18.6 (−24.8 to −12.4)	0.521
ΔPmax, ms	−12.4 (−19.5 to −5.3)	−8.2 (−13.8 to −2.6)	0.285
ΔPWD, ms	−18.0 (−38.5 to +2.5)	−12.0 (−30.1 to +6.1)	0.482

[a] Independent-samples *t*-test or Mann–Whitney U test as appropriate. This post-hoc stratified analysis was not specified in advance, was not included in the multiplicity-controlled family, and is reported with nominal *p*-values. AF, atrial fibrillation; PWD, P-wave dispersion.
